# Medicinal plant use in two Tiwi Island communities: a qualitative research study

**DOI:** 10.1186/s13002-019-0315-2

**Published:** 2019-08-16

**Authors:** Adam Thompson, Gemma Munkara, Marie Kantilla, Jacinta Tipungwuti

**Affiliations:** 1 Menzies School of Health Research, PO Box 41096, Casuarina, NT 0811 Australia; 2 Strong Women’s Group, Wurrumiyanga (Tiwi Islands), Tiwi Land Council, PO Box 38545, Winnellie, NT 0821 Australia

**Keywords:** Medicinal plants, Bush medicine, Tiwi, Wurrumiyanga, Ethnography, Qualitative, Medical anthropology

## Abstract

**Background:**

Traditional medicinal plants are still used today in many Aboriginal communities across Australia. Our research focused on the contemporary use of such plants in the two communities within the Tiwi Islands, Wurrumiyanga and Pirlangimpi.

**Methods:**

This qualitative research project performed a video ethnography, community interviews, and a trial intervention to better understand the extent to which these plants are still used throughout the community and how they may be used more in the future.

**Results:**

We found that several plants are still used predominantly as medicinal washes to treat skin disorders and/or as a tea to treat congestion associated with cold and flu. Those plants that are commonly used are found near to the community in large amounts and are recognized as being both safe and effective.

**Conclusions:**

Within the community, it is the elder women who remain most knowledgeable about these plants and continue to make them for their families. However, there are many families who no longer know how to make these traditional medicines though they express a desire to use them. Therefore, it would be beneficial to have a central location or method to produce traditional medicine for the community—a bush pharmacy.

## Background

The Tiwi Islands lie within the Northern Territory of Australia approximately 90 km north of Darwin where the Arafura Sea joins the Timor Sea. The islands were separated from the mainland by rising sea level at some point 7000–15,000 years ago [1]. The mythology of people living on the islands, who refer to themselves as the Tiwi, records this sea level rise in the story of Mudangkala, an old blind woman who was the original ancestor of the Tiwi people who crawled from the Australian mainland while water followed behind her to create the islands [2, 3]. The Tiwi are believed to have remained in relative isolation from mainland groups ever since the islands became separated and developed a culture relatively distinct from mainland Aboriginal Australians [3]. Tiwi people have unique cultural practices including major ceremonies such as the kulama, a yam harvest ceremony during which male initiation rites occur, and the pukamani burial ceremony. Both the didgeridoo and concepts of sorcery were not present within Tiwi culture until very recently and are still not recognized as aspects of the ancestral culture. Flora and fauna on the islands are similar to mainland Australia but with a higher proportion of monsoon forest ecosystem and mangroves as the islands receive the highest rainfall in the Northern Territory (about 2000 mm in the northern sections of the islands [4]. Some similarities with mainland groups do exist in traditional ecological knowledge due to similarities between environments and a common ancestral culture prior to the separation of the islands. Since the 19th century, contact between the islands and the mainland has gradually increased facilitating the sharing of cultures. Today, regular travel between the islands and the mainland occurs with several small plane flights every day. Nonetheless, the Tiwi people still see themselves as a unique culture and people. Today, the Tiwi island population is composed of approximately 2500 people with about half the population living in the largest community of Wurrumiyanga (aka Nguiu) on Bathurst Island and roughly 300–500 people living in Pirlangimpi (aka Garden Point, Pularumpi) and Milikapiti (aka Snake Bay) on Melville Island.

Early descriptions of Tiwi islanders gave special attention to the high quality of their physical health [1]. Tiwi believe that, prior to European arrival, every Tiwi person was expected to live into old age and their life would only be cut short by fighting or accident [5]. At the time of European contact, the Tiwi still lived predominantly in small bands that spread across the landscape for the majority of the year. Each day, the women and men would venture out in groups of 2 or 3 to collect food for the day. Older women were acknowledged as the primary food collectors who taught younger women the skills for foraging the myriad of vegetable foods available in the bush [6]. Much of this collection occurred within the mangroves. Several historic records document how polygamy was asserted to be a necessity of life for men, since, in order to eat well, a man needed many wives to collect food with the older wives being the most important because of their accumulated knowledge [1]. Tiwi men claimed that if a man had only one young wife, as the missionaries encouraged, he would go hungry. Those men that did convert to Christianity were still seen to have many older women in their family group who were related to him to provide food [7].

Goodale writes that any disease was viewed as the result of failing to carry out ceremonies in the proper manner [6]. Simeon [5] describes how Tiwi rituals require careful attention, and any mistake in following the strict decorum may lead to torni/tarni, a spiritual sickness. In 1954, when Goodale observed the kulama ritual, its stated purpose was more about preventing sickness and less about harvesting yams and male initiation. This is likely different than the ceremonies role prior to the introduction of Western diseases. Traditionally, such ceremonies were the only time when large amounts of people would come together and therefore represented the few opportunities for disease to spread rapidly. In contrast, mainland Aboriginal Australians often attributed sickness to the work of sorcerers within enemy groups. The concept of sorcery was introduced to the Tiwi islands with the arrival of Iwaidja mainlanders in the 19th century, but it is still recognized as an introduced concept. The introduction of epidemic disease by Iwaidja and Europeans likely acted to validate these claims [6]. For example, mainland magic was believed to be one of the causes of leprosy [8].

Johnson [8], writing in the 1980s, disagrees with Goodale and believes that illness was more often attributed to yirrankimi, the feeling of shame that results from being refused something. This may be the result of changes in settlement which placed extended families in close contact year round creating an overbearing burden to share that had not been experienced before [9]. By the 1960s, when official census records began, this settlement pattern had changed completely to a permanent sedentary life within large communities facilitated by the influence of missionaries [10]. This transition occurred for many Aboriginal people across the Northern Territory as well. These larger communities made the provision of health and educational facilities easier and led to the subsequent decreases in child mortality [11] and increases in education. However, they have also led to problems associated with the loss of cultural identity and increases of infectious disease. Thus, concepts of health seem to have changed even over the past century from the introduction of western disease impacting the role of ritual protocol, introduced concepts from mainland groups such as sorcery, and the crowding of communities that placed greater importance on social interaction.

Still today, the Tiwi people of Wurrumiyanga practice their two major traditional ceremonies: kulama and pukamani. The kulama occurs annually and is a 3-day ceremony in which a certain species of yam (*Dioscorea transversa*) is harvested and given as an offering in connection to an early creation story. This species of yam is only eaten for this ceremony and is toxic unless prepared in a specific way. While the ceremony was filmed in recent times, the video recording was for documentation purposes only, was not distributed, and required several years of negotiation to complete. Many of the traditions around the ceremony, including its secrecy, are still maintained. The ceremony itself is only for men to take part in and is associated with male initiation ceremonies. However, women artists create paintings that tell the creation story associated with the ceremony as well as paintings that depict the yam itself, which is one of the few plants regularly painted. It is clear that people are very proud that the ceremony still takes place and the knowledge around it is an essential part of their art.

However, by comparing past ethnographies and questioning more deeply into the nature of the kulama, it becomes clear that many aspects of the ceremony have changed over time [12]. Traditionally, the kulama ceremony was associated with a complex initiation process involving six distinct grades that represented a formal process of schooling [13]. Ceremonies occurred outside the primary kulama ceremony as part of this teaching process as well as protracted periods of isolation for those undergoing initiation. Historically, the completion of these stages of initiation was necessary before marriage was allowed. After completing all the stages, initiates had to be adept enough at the old language in order to improvise new songs within the ceremony according with traditions. To perform badly was seen as dangerous. There are only a few members of the younger generation that are confident enough in the old language to improvise new songs. The elders who can perform this aspect of the ritual are dying off. This is seen as the result of the introduction of Western school system by early missionaries and the subsequent devaluing of ceremonial life. As a result, the kulama ceremony is now performed more to preserve a lingering tradition than as a living culture. At present, the churches in Tiwi have come to embrace traditional culture and work to incorporate traditional ceremonies into their own work, for example, the Catholic Mass will be performed at a funeral as well as the traditional pukamani funeral ceremony which is still performed today. This ceremony features the funeral pole that is a major feature of Tiwi art and a symbol of their culture. It is unique to their culture and sets them apart from mainland Aboriginal groups. The Tiwi are quick to address their culture as being different from mainland Aboriginal people while still respecting those mainland cultures. Thus, the Tiwi way of using traditional medicine is still the Tiwi way even if it overlaps with those on the mainland.

It is clear from these examples that Tiwi traditions are undergoing a process of change. Within this context, the Strong Women’s Group have taken an active role to conserve traditions in a new form. They have created new songs that fulfil the traditional roles of song for teaching, healing, and passing down knowledge in a new style that is still uniquely Tiwi [14, 15]. These new songs are not seen as a replacement for old traditions but more as an addition. For the purposes of the current study, it became clear that they would be best to work with in order to re-package traditions around medicinal plants for the modern Tiwi culture.

### Tiwi ethnobotanical knowledge and traditional food systems

The use of medicinal plants in Tiwi communities has been documented over the past 35 years [8, 16–18]. The majority of these medicinal plants may be used in the form of a wash to bath the body with fewer medicines being ingested. Prior to the development of modern plumbing technology, clean water was less available and more precious. Natural water holes provided the opportunity for bathing as long as they remained unpolluted. These waterholes are commonly associated with spirits that can cause sickness if disturbed. They are also the sites of sacred rites of passage. In Tiwi, this is evident in ampiji, or rainbow serpent, who lives in a lake at Mangantu on the southwestern side of Bathurst Island, but is also associated with all natural water features including rain [8]. In such conditions, it may be advantageous for bathing to occur less frequently but with the use of medicinal plants to act as anti-bacterial agents to cleanse more deeply. Laboratory testing of many of the medicinal plants used by the Tiwi have found them to have anti-microbial properties [16]. Tiwi medicinal plant knowledge reflects a sophisticated understanding of the use of plants as cleansing agents specific to different conditions (chest infection, cold, flu, skin disorders). Medicinal plants are used as a wash in similar ways by mainland Aboriginal Australians across the Northern Territory [18, 19], and therefore, these traditions may extend back in time to a common ancestral culture prior to the separation of the islands.

Traditionally the use of such plants are believed to have occurred routinely in the same manner that food plants were collected. When plants were in season, they were collected and used. Traditionally, choices on diet were limited. People ate what the land provided and trusted that what the land provided was healthy. Most traditional medicines utilize the leaf or inner bark of the plant and are therefore available all year round. In this way, the collection of traditional medicine is in many ways analogous to the collection of traditional food plants. Often the two are viewed as the same. The process of going to the bush to collect these plants is the same as are the obstacles. A recent study conducted across 20 Aboriginal Australian communities showed that traditional foods were frequently consumed across all communities [20]. The study cited the need for comprehensive assessments of traditional food systems in order to develop ‘targeted strategies to ensure sustainable access and increased consumption of traditional foods’ (page 4). The same recommendation could be made for traditional medicine. In her literature review, Brimblecombe shows that the proportion of traditional foods within the diet may vary widely between communities [21]. As yet, few studies have been performed on the frequency of use of traditional medicines in Aboriginal Australian communities [22].

## Methods

**Fig. 1:**
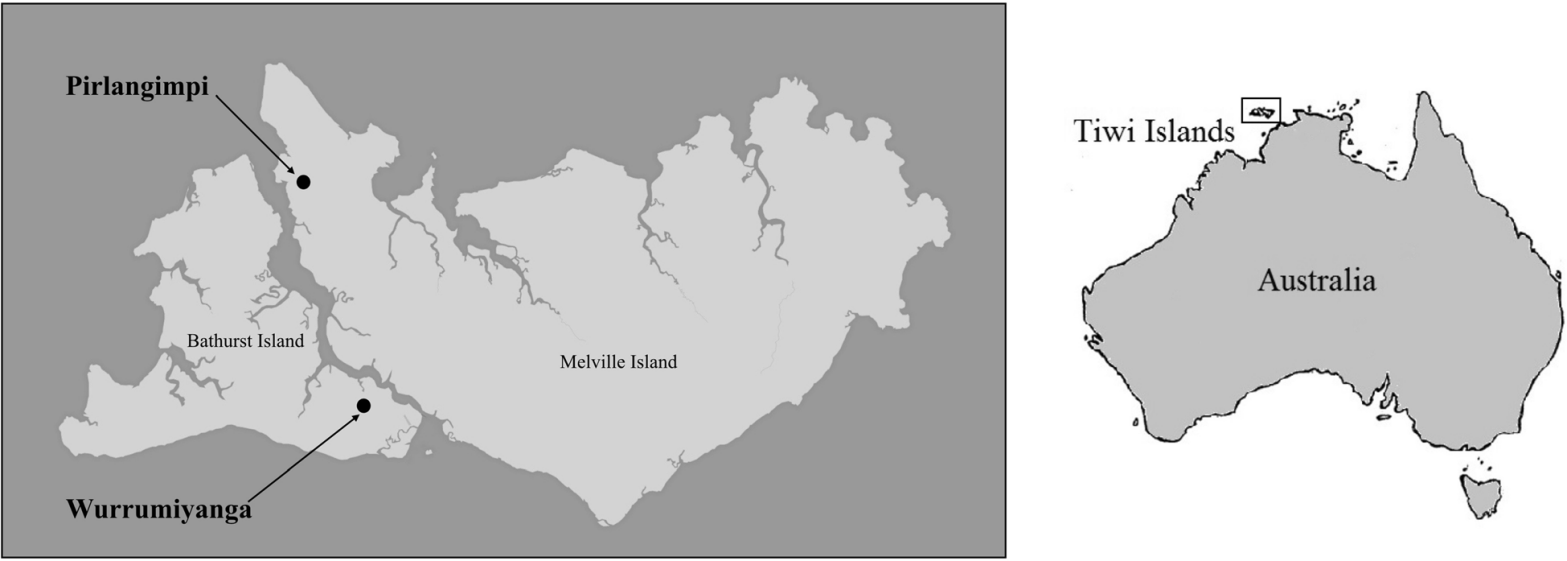
Map showing location of Tiwi Islands, Wurrumiyanga and Pirlangimpi

Our project hoped to answer some basic questions on the extent to which medicinal plants are still used today in two Tiwi communities, Wurrumiyanga and Pirlangimpi (Fig. 1). Both communities have modern facilities including well-stocked health clinics, shops, and schools, and both are located on the coast within Apsley Strait. The difference between the two is subtle. Wurrumiyanga is larger, is further south on Bathurst island, and may receive slightly less rainfall, while Pirlangimpi is smaller and is found further north on Melville Island with slightly more rainfall. Within the context of these modern communities, we wanted to understand the role that medicinal plants still held.

The project began with a focused video ethnography involving field trips with those within the community recognized as the appropriate knowledge holders to collect and prepare plants within the creation of a short documentary film to provide to the community. Validation for the use of video ethnography as an appropriate method has been previously published [23]. This ethnography provided an understanding of the medicinal plant knowledge still retained today and the accessibility of medicinal plants, and documented the complete methods of preparation. Field trips were performed in the wet and dry seasons to compare seasonal availability.

Following the video ethnography, interviews were performed with people throughout the community. Local institutions specifically the Red Cross, women’s centre, aged care centre, Strong Women’s Group, children’s care centre, wellness centre, Tiwi Land Council, and the clinic were all approached to provide participants. In this way, the perspectives of these local institutions and connections between them could be mapped out as well. Subsequently, a snowballing sampling strategy was utilized whereby participants and contacts could suggest other potential interview participants. This method meant there could be a bias towards people who were knowledgeable of traditional medicine and those related to previous participants. Individual’s totem and kinship group were recorded to assess the sample distribution. Semi-structured interviews were conducted with 36 participants in Wurrumiyanga and 9 participants in Pirlangimpi. Informed consent was obtained before each interview after explaining the purpose of the study and how the information provided would be used. The participation of local co-researchers who could communicate in Tiwi ensured informed consent was obtained.

People were commonly found in groups of family members composed of multiple generations allowing questions to be asked directly about differences in knowledge between generation and gender within individual families. Attempts were made to interview at least two members of the same family of different generations. In cases where larger groups were present, interviews focused on two participants to ensure information were recorded in an orderly fashion allowing interview participants to corroborate what they were saying with other family members of the same generation and gender. Age and gender were recorded for each participant. This allowed our study to make some general conclusions about the use of specific plants across generations and within gender categories.

Interviews began by asking people the most recent instance in which ‘you’ personally used traditional medicine or made traditional medicine for someone else. Care had to be taken that interview participants understood that the word ‘you’ referred to them as an individual and not to their extended family, the community, or the Tiwi people in general. As a collectivist society, there is a tendency for concepts of ‘you’ to extend to larger groups but people were able to understand what was being specified. This is one reason why a simple survey form would not work and interviews were required. Further questions looked at when people first used traditional medicine, who prepared it, and differences between wet season and dry season availability. Interviews were audio recorded and transcribed. Data was collated and analysed using the NVivo software program.

Following the completion of interviews, a trial intervention was performed. Two types of traditional medicine were made and shared with anyone in the community who wanted to use them. This method allowed us to gather information from people who did not regularly use traditional medicine but who were interested in trying it.

## Results and discussion

### Results of focused ethnography

The wet season which occurs from November to April is not a good time to travel outside the community to the bush. The dirt roads are washed out, and it is recommended that one use a four-wheel drive vehicle. The grass grows tall, and it is hard to see snakes which can be poisonous. Saltwater crocodiles are also nesting at this time, and any trip near water is inadvisable. These difficulties are relieved in the dry season (May to October) when the roads dry up, the grass is burned, and the crocodiles move to deeper river systems and are no longer mating. On our initial trip, certain areas in the bush were pukumani, or taboo, according with traditional funeral rites and we were unable to travel to them. Nevertheless, our project was interested in demonstrating what plants were available throughout the year and so collecting plants in the wet season was imperative. Despite limited access, eight plants were identified near to the community from three environment types: the eucalypt forest, dry rainforest, and coastal sand flats.

Within the eucalypt forest close to the community, the most commonly available plant was jimijinga (*Persoonia falcata*). Its leaves are boiled as a tea to treat coughs, flus, and congestion or may be used as a wash, or the steam from the boiled infusion may be inhaled. Also available within this ecosystem was tokapunga (*Ficus* sp.), kanuli (*Planchonia careya*), timirarringa (*Eucalyptus miniata*), and miyaringa (*Pandanus spiralis*). Tokapunga, kanuli, and timirarringa could be boiled as a wash to treat skin sores. The tokapunga leaf is used while the inner bark is used for kanuli and timirarringa. Miyaringa is eaten to settle the stomach.

Within the dry rainforest behind the mangroves, we found jikiringi (*Alphitonia excelsa*) also known as soap tree. The leaves are crushed, mixed with water, and used like soap. This plant is also known by many other mainland Aboriginal groups. It was acknowledged that this plant is mostly used by bathers in the dry season when they are swimming at the waterhole where soap tree grows abundantly within the wet rainforest. Being close to the community, it was easier to acquire store-bought soap than to collect soap tree leaves. Tokapunga could also be found here in greater quantities.

In the coastal beach, rokuni (*Ipomoea pes-caprae*) grows abundantly. A few handfuls of leaves are broken roughly and boiled in a pot of water, and the resulting liquid is used to treat skin sores and rashes once it has cooled. After demonstrating the manner of preparation, it was carefully applied to the skin sores of a baby who could not yet walk, approximately 6 months of age. Care was taken to avoid the eyes and mouth and to only apply the wash to the sores using a new cloth that was purchased at the store. The baby showed little distress from the application. The next day, the sores did in fact look better. It was recommended that the wash be applied to the sores three times a day. The boiled infusion would last 3–4 days if kept in the refrigerator. Also available on the beach was tarripilima (the germinated seed of *Rhizophora stylosa* or *Rhizophora apiculata*). These were collected from the high tide line where they washed up and their name refers to their source ‘from the sea’. They too could be crushed and boiled to treat skin sores and scabies in the same manner as rokuni. Their availability varied throughout the year.

During the collection of plants, several practical factors presented themselves. The abundance of plants and ease of collection effected what was collected. Rokuni, tarripilima, and jimijinga were the most abundant and easily accessible within Wurrumiyanga. Timirraringa and Kanuli were more abundant in Pirlangimpi and were preferred (Table 1). This collection thus showed that several plants could be obtained in the wet season to treat both skin sores and general sickness.

**Table 1:** Medicinal plants commonly used in Wurrumiyanga and Pirlangimpi

Plant—Tiwi name (scientific name)	Part of plant used	Environment where plant is found	Condition treated	Method	Voucher no.
Rokuni (*Ipomoea pes-caprae*)	Leaf	Coast	Skin	Wash	A.T. Thompson 014 (DNA) Wurrumiyanga
Tarripilima (*Rhizophora stylosa*)	Hypocotyl	Coast	Skin	Wash	A.T. Thompson 015 (DNA) Wurrumiyanga
Timirraringa (*Eucalyptus miniata*)	Inner bark	Woodland	Skin	Wash	A.T. Thompson 016 (DNA) Wurrumiyanga
Jimijinga (*Persoonia falcata*)	Leaf	Woodland	Congestion	Wash, tea, and/or inhaled	A.T. Thompson 017 (DNA) Wurrumiyanga
Tokapunga (*Ficus opposita*)	Leaf	Woodland	Congestion	Wash and/or inhaled	A.T. Thompson 019 (DNA) Wurrumiyanga
Kanuli (*Planchonia careya*)	Inner bark	Waterhole/woodland	Skin	Wash	A.T. Thompson 022 (DNA) Wurrumiyanga
Jikiringini (*Alphitonia excelsa*)	Leaf	Waterhole/woodland	General wash	Wash	A.T. Thompson 018 (DNA) Wurrumiyanga A.T. Thompson 021 (DNA) Wurrumiyanga
Miyaringa (*Pandanus spiralis*)	Leaf base	Waterhole/woodland	Stomach	Ingested	A.T. Thompson 020 (DNA) Wurrumiyanga
Miparriyi (*Livistona humilis*)	Heart of the palm	Waterhole/woodland	Stomach	Ingested	A.T. Thompson 023 (DNA) Wurrumiyanga

In the dry season, road access improves dramatically allowing community members to travel regularly to the waterholes and mangroves. It is this time of the year when people perform bush holiday, camping on the traditional lands for 3–4 weeks. This is an important time for people to practice their traditional culture and connect with their traditional homelands. An elder claimed that in the past, people would go ‘bush holiday’ for 3 months or longer but had gotten shorter over time. Nevertheless, while the duration of time people spend may be shortening, it remains an important event for all people and many stories are told of experiences living in the bush. It is at this time that people may experience bush foods that are found further inland. While these foods represent a small portion of the overall diet, they are important culturally to the identity of people. They include many varieties of plants such as plums and apple available early in the dry season and sugarbag, honeycomb made by the native bees within tree hollows. People also described the health benefits of bathing in running water within the creeks where jikiringini is abundant.

Certain plants such as jikiringini (*Alphitonia excelsa*), kanuli (*Planchonia careya*), and Miparriyi (*Livistonia humilis*) grow abundantly within the wet rainforest around the waterholes. These inland waterholes are free of crocodiles and provide a traditional bathing area for bush holiday. Jikiringini was said to be used during this time by all people. The white inner heart of palm of Miparriyi (*Livistonia humilis*) is an important food source which is roasted and is said to be good for the stomach. The inner bark of kanuli is used to treat skin disorders. Mangroves could also be more easily accessed this time of year where many important iron-rich foods may be found including a longbum, mussels, and mangrove worm of which there are two types: yurli and wakatapa. Wakatapa, or cheeky mangrove worm, should not be eaten raw as it irritates the throat and causes it to burn. However, it may be boiled and drank to act as a decongestant for colds and flus.

Two plant foods found in the bush that were mentioned as being particularly healthy are wupuna (*Amorphophallus galbra*) and minta (*Cycas armstrongii*) [17]. Both of these are naturally toxic and must be processed to be transformed into edible food: wupuna is cooked in the ground for at least a day, and minta is pounded and left in running water to leech the poison out for 3 days. The lengthy process means that they are rarely made today, and many said it had been 10–15 years since they were last made though there was interest in renewing the practice. It is the consumption of these two foods that many people associated with the health of their ancestors. Culturally, there is special significance to the process of transforming toxic plants into edible foods. The kurlama ceremony occurs annually in the dry season and is the only time in which the naturally toxic kurlama yam is consumed. The ability to transform something that causes death into something that creates life appears to be a central cultural motif. The importance of funeral ceremonies may be included as they transform the death of an individual into an opportunity for all those connected to the deceased to reinforce their bonds as a social group [24]. In this way, the declining use of wupuna and minta due to the shortened bush holiday may be associated with the decline of traditional culture.

### Results of community interviews

Women throughout the community had a strong knowledge of the medicinal plants collected within our study, particularly plants used as medicinal washes. These could be divided into plants used for skin conditions (tarripilima, rokuni, timirraringa) that were mostly used on children and a single plant jimijinga) used for symptoms of cold and flu such as runny nose which is called jikaputi in the local language for children and adults. Women tended to report first making these medicines once they had their first child. At this point, they became responsible for making traditional medicine for their family. They were assisted by their parents and close family members. Once women become grandmothers, they take on greater responsibility as the authority in their family on the proper method of making traditional medicine. Most all women of all generations reported first seeing their mothers or grandmothers make traditional medicine from a young age, commonly reported as being from the age of 6, but it was not until they became mothers that they made it themselves.

Adult men rarely reported making traditional medicines used for skin conditions as these tended to be used on children. Commonly, they said the last time they used such medicines was when they were young teenagers roughly 10–14 years of age and that their mothers and grandmothers made the medicine for them. Beyond this age, men are no longer under the care of the women in the family and must take care of themselves with the assistance of other men. Men commonly reported bathing in saltwater as the treatment of any skin conditions though one older man reported making tarripilima to treat infection on his hands. Men did report making jimijinga either for themselves or male family members, but this tended to be done in more serious conditions once a person was bed-ridden.

Men instead tended to focus on traditional foods that were hunted or collected from the bush which were seen to be especially nutritious foods with specific health-giving properties. For example, wakatapa, cheeky mangrove worm, was used more often for cold and flu than jimijinga. Men would talk about saltwater foods such as fish, turtle, or dugong, which they provided to their family. Both men and women talked about the benefits of eating ‘longbum’ shellfish which was always stated to be good for blood because it is iron-rich. Other shellfish were also mentioned in this category such as mussels and oysters. Mangrove worm, of which there are two types, was also commonly mentioned by both men and women of all generations. One type yurli was said to be good for breastfeeding women, while the other wakatapa was good for people with cold and flu. The muddy secretions from the mangrove worm were also commonly mentioned as being good for certain skin conditions.

Children from age 0–14 regardless of gender are cared for primarily by the mother but also by the female members of the extended family who provide traditional medicine. Jimijinga was acknowledged to be safe for children of any age though it would be given in different forms. For breastfeeding babies, the mother would drink jimijinga tea and the baby would receive the benefits through the breastmilk. It was also stated that the mother could chew the leaf and gently spit the juice into the child’s mouth. Children could be bathed in jimijinga once they were a bit older, ‘running around’, or inhale the vapors of the boiled medicine. Rokuni, tarripilima and timirraringa could be used on children as long as care was taken to avoid the face with preference for sores to be washed directly. Once a girl reached womanhood represented by marriage and the birth of her first child, she became responsible for deciding to use traditional medicine and creating it herself. This responsibility was acknowledged by women who said they would only make traditional medicine for someone if they were asked. When boys became young men, they become responsible for their own care and must make their own medicine or receive assistance from male relatives in which case jimijinga and saltwater are the two medicinal washes most commonly used. These changes into adulthood begin to occur when brothers and sisters are no longer allowed to play with each other and avoidance relationships develop.

### Perception of medicinal plant use

In Wurrumiyanga, there is a health clinic which provides general care, a well-being centre next to the clinic that address mental health and holistic health, a women’s centre that can address women’s health issues, and an aged-care centre. All four of these centres expressed support for the use of medicinal plants in general but made few specific recommendations. This was true of the clinic in Pirlangimpi as well. A flip chart at the well-being centre recommended rokuni for scabies, and a community-made publication at the women’s centre recommended tarripilima, rokuni, tokapunga, or timirraringa for scabies and skin sores. Both of these provided simple instructions on the processing and use of these plants. However, it was generally acknowledged that Aboriginal health workers could provide more detailed information and links to strong women and elders in the community who knew more. This deferment was likely done out of respect for the cultural knowledge of these individuals. However, it results in a separation of traditional medicine from these western institutions.

The fact that traditional medicinal plants are embraced amongst these centres is significant. Additionally, the local school took children out into the bush and taught them about many of these plants as well. Nevertheless, the use of such plants still depends on individuals and/or their families to collect and prepare traditional medicines on their own. This task has its barriers. Sufficient knowledge and confidence may be held by only a few elders in the family who may need the use of vehicles to access areas of the bush where the plants are found. Confidence in traditional medicines is waning amongst the younger generation, and so motivation becomes a factor as well. At times, simple equipment such as clean pots for boiling medicine, firewood, stove, or an axe may also be an issue. The value then of having a central place where traditional medicines that are recommended by health centres could be made available then becomes clear. Such a location would increase the treatment of ailments such as skin sores and relieve the burden of minor ailments from the clinic.

Those that continued to use medicinal plants often stated they used them because they felt they were safer. The clinic medicine was seen to be stronger while medicinal plants were believed to have a milder effect that was safer for babies and young children. While no plants were said to be used in combination nor was traditional medicine and clinic medicine used simultaneously, some people reported using different medicines on alternating days. For example, the treatment prescribed by the clinic for scabies was to apply lycreme over the entire body once and then wait a week and re-apply. During the week in between treatments, some people said they would use traditional medicines such as tarripilima.

The demand for traditional medicinal plants became clear once the boiled infusion was made available. Following field collection of plants, one species of plant would be boiled up to demonstrate the manner of preparation and use. This process took place within the strong women’s area under a couple of large trees next to the open-air church. The area beneath the trees was a place for all sorts of people to congregate: strong women, young women and their babies, a few elder men, and card players. While the medicine was being prepared, little attention was paid by others to the process. However, once it was complete and the medicine was ready, most everyone present would become involved in its use. The preparation of rokuni was followed by the careful cleaning of a baby’s sores under the attention of at least seven women. The preparation of jimijinga was followed by the sharing of the boiled tea with at least eight people and the distribution of the remaining leaves. In both cases, people who had never prepared or used the specific medicinal plants were present and tried them for the first time. These examples demonstrated that there was a great demand for traditional medicine when it was available. Less attention, however, was given to the preparation process.

The strong women generally acknowledged that there was a lack of interest within the younger generation. This seemed to result from the dichotomy placed between western clinic medicine and the traditional medicine of elders. Many expressed concern that the two forms of medicine may interact in a harmful way. While it was the general belief of health staff that the two forms of medicine would not likely interact or would rather be mutually beneficial in most circumstances, it was difficult to say that this would be true in absolutely every case given the duty of care required. This concern for cross-interaction then led people to feel they must choose one or the other form of medicine. Having grown up in the community, the younger generation had decided to use the clinic medicine which was close at hand. Several strong women expressed that the younger generation questioned whether the medicinal plants actually worked. Traditionally, these plants were used based on faith in the knowledge of elders and personal experience with their results. Today, greater confidence is given to the authority of the clinic. Under these circumstances, it may be necessary for the clinic to take a more involved approach to the recommendation of medicinal plants if they are actually to be used rather than simply referring to the recommendation of Aboriginal health workers and community elders. Laboratory testing of plants has taken place [16] and is continuing which could allow clinics to take a more involved approach.

### Results of bush pharmacy trial

For the trial intervention, we created two types of traditional medicine, one for jikaputi (coughs and flu) and another to treat skin conditions, and provided these to anyone in the community who wanted to use them. We called our intervention a bush pharmacy. In Wurrumiyanga, we prepared jimijinga and tarripilima, while in Pirlangimpi we prepared jimijinga, timirarringa, and kanuli. Decisions on which medicine to make were made based on availability and common knowledge. It took about half a day’s work to create each of the medicines. They were then stored in the refrigerator and distributed the next day. To distribute the medicine, we drove around the community stopping at locations where people were known to gather around and visiting people who it was known would benefit from the medicine. At each location, it was common for a group of 4–5 people to use the medicine and discuss its benefits.

In total, at least 30 people used the jimijinga we provided. It was most commonly used by older people who more often complained of coughs or congestion. It was also used more often by women, though this may have been the result of bias as the local co-researchers supplying the medicine were women and felt more comfortable approaching. Future studies should employ men to distribute the medicine as well. The common response from people was that they had not had jimijinga for a long time, though they remembered it and described it as ‘the same taste as when I was a child’. One elderly person said it had been a long time and was not sure if his body would react differently to it, but after a drink and some light exercise, he coughed up some phlegm and felt it was working well enough that he requested a full bottle. Another lady insisted on blessing the medicine prior to drinking and insisted others do the same. People visiting from other communities appreciated having the medicine offered to them as they did not feel capable of collecting the plants themselves as well as those with mobility issues. Others tried it for the first time and were told enthusiastically about it. We thus found that the distribution of the medicine created the opportunity to see who had not used it before and share information about it.

Tarripilima, timirarringa, or kanuli were provided to mothers to treat children’s skin sores. Some mothers were familiar with its use, and treatment was applied directly to a total of 8 babies according with their mother’s wishes. The recommended treatment is for the baby to be bathed from the neck down. Because mothers preferred to bath their babies at home, we provided bottles of the medicine, but washed visible signs of scabies directly with a new cloth on site to ensure treatment was provided and instructions were given on its use. The babies that were treated commonly had initial signs of scabies on their hands and feet which had not progressed far enough for them to seek treatment at the clinic but was visible enough to be worth treating with the traditional medicine we provided. This experience showed that this intervention could provide early treatment before the condition became worse. In several cases, other family members were present including the fathers who were very supportive of the use of the medicine and provided their own stories of being bathed with traditional medicine as young boys or being instructed in the creation of traditional medicine by their grandfather.

This brief trial of a mobile bush pharmacy proved quite successful with many promising benefits. The distribution of traditional medicine could target those known to need it that could not create it themselves, including elderly people, and those staying at the aged care centre. It could provide treatment early without people needing to go to the clinic which proved especially valuable for babies with scabies. Most importantly, it provided the opportunity for people to discuss traditional medicine, how it had been used in the past, and to encourage those who had not used it before to try it. These benefits resulted not just from creating and providing medicine from the plants easily accessible in the community, but also from distributing the medicine from a vehicle that allowed us to approach people throughout the community. Plans are now underway to continue to provide a mobile bush pharmacy on a regular basis.

### Differences between communities

Pirlangimpi and Wurrumiyanga are very similar in many respects: they have a similar origin as a Catholic mission, both are located on the coast with waterholes a short drive away, and both have the same Tiwi culture. As one Tiwi elder said, ‘Everything’s the same in these two islands, wherever you go’. Performing the same methods provided the same results. The same plants were used in the same way. The same gender differences could be found. The one exception was a preference for timirraringa and kanuli both made of inner barks instead of rokuni and tarripilima which are found on the beach. The reason for this difference is likely the fact that large trees could be found closer to the community, a feature also noted by other sources [4, 25] and were more easily accessible whereas in Wurrumiyanga their collection required a longer drive. In both communities, these inner barks were said to be more effective.

### Comparison of Tiwi medicinal plants to mainland aboriginal Australians

A comparison of the medicinal plants used in Tiwi to ethnobotanical records compiled thus far for mainland Aboriginal groups shows both differences and similarities. Some Tiwi medicinal plants appear to be quite unique. While the fruit of *Persoonia falcata*, also known as milky plum, is recognized and eaten by mainland groups, the use of the leaves as a tea may be unique to the Tiwi islands. Elsewhere, a decoction from its inner bark is used as an eyedrop [18]. Likewise, while the medicinal use of the bark of *Rhizophora apiculata* [26] has been recorded outside of Australia, the use of its hypocotyl may also be unique. The specific use of *Eucalyptus miniata* may be unique; however, the inner bark of other Eucalypt varieties is used by mainland Aboriginal Australian groups in the same way such as *E. microtheca*, *E. pruinosa*, and more commonly *E. tetradonta* [18]. For other Tiwi medicinal plants, there are only a few other records of similar use. The leaves of *Ficus opposita* are also used by people in the community of Milingimbi, and *Livistona humilis* and *Planchonia careya* are both used in the same way by Iwaidja speakers of Minjilang [18, 27]. Historically, Iwaidja speakers travelled to the Tiwi islands to work in buffalo hunting camps [1] and the common use of these plants may have come from the sharing of knowledge at this time. Other medicinal plants are known to be used by a many different mainland Aboriginal groups. The use of *Alphitonia excelsa* as a form of soap has been recorded for several other mainland groups [18, 28, 29], while the ripe seed pods or leaves of different *Acacias* are used in most other groups where *Alphitonia* is not [30–34]. Both *Ipomoea pes-caprae* and *Pandanus spiralis* are commonly used by many different groups [18].

## Conclusions

While the records of previous ethnobotanical reports provide a list of 32 medicinal plants and 3 non-plant substances used as a form or traditional medicines, the focused ethnography performed by our study found that modern use focused on only 9 medicinal plants and 2 non-plant substances, green ant nest and mangrove worm. While ethnobotanical records serve as a repository of knowledge, they are not necessarily a complete representation of the current use of medicinal plants. One should expect that some plants would be used more predominantly than others, specifically those in the greatest abundance and easiest obtain, which are known to be safe and effective. By surveying the community through interviews, we were able to focus on the most commonly used medicinal plants used for two different conditions, coughs/congestion and skin disorders.

Those medicinal plants that were used were most commonly made by women to treat children’s skin disorders. Men reported using Jimijinga but tended to focus more on the use of bush foods such as wakatapa, cheeky mangrove worm. The younger generations also seemed to be using traditional medicines less. For these reasons, there is a perceived potential for a central location where traditional medicinal plants could be processed and provided to the community. This would allow a standardized approach to be established that would allow for the clinic and other community centres to make specific recommendations. The creation of such a place has been done in other parts of Australia such as the Akeyulerre Healing Centre in Alice Springs [22]. Some community members may find it more comfortable to attend these healing centres where traditional medicines are available than to go to the clinic. In both communities, participants described how traditional medicine was once used within the clinics to treat children’s skin infections.

While culture on the Tiwi Islands has changed over the past 100 years, traditional medicinal plants still serve an important function for the health of the community. Maintaining traditional knowledge does not require people to return to an archaic mode of life. Instead, it involves developing new modern methods that utilize traditional knowledge in new ways. These new methods must be community-driven. In which case, the form they take is often unpredictable. Their creation does not necessarily come from lofty planning and foresight. Rather, they develop out of practice—from people going out to the bush collecting plants and making medicine, from people telling stories of the plants they have used in the past, and from the hopes for what will come in the future.

## Data Availability

All publicly-available data generated or analysed during this study are included in this published article and in the short documentary films, Bush Medicine in Wurrumiyanga (https://vimeo.com/312431196) and Bush Medicine in Pirlangimpi (https://vimeo.com/315569215). Datasets generated from community are not publicly available for reasons of privacy but may be requested from the lead author and are stored at Menzies School of Health Research.
